# Chromosome-Level Sex-Specific Genome Assemblies of *Onthophagus binodis* Provide Insight into Scarab Sex Chromosomes

**DOI:** 10.1093/gbe/evag023

**Published:** 2026-01-29

**Authors:** Erica M Nadolski, Yongsoo Choi, Joshua A Jones, Armin P Moczek, Phillip L Davidson

**Affiliations:** Department of Biology, Indiana University Bloomington, Bloomington, IN, USA; Department of Biology, Indiana University Bloomington, Bloomington, IN, USA; Department of Biology, Indiana University Bloomington, Bloomington, IN, USA; Department of Biology, Indiana University Bloomington, Bloomington, IN, USA; Department of Biology, Indiana University Bloomington, Bloomington, IN, USA; Department of Biological Sciences, Mississippi State University, Mississippi State, MS, USA

**Keywords:** dung beetle, Coleoptera, Y chromosome

## Abstract

*Onthophagus binodis* is a coprophagous scarab beetle native to southern Africa. This species and many others in the tribe Onthophagini have been introduced to farms across multiple continents in the context of cattle pasture management efforts. The ecosystem services provided by this species, along with the amenability of comparative developmental and evolutionary studies in this clade, contribute to its role as an emerging insect model system. Here, we present sex-specific chromosomal-level genome assemblies for *O. binodis* generated from a combination of PacBio long reads and HiC chromatin conformation sequencing. The completeness of the 950.5 Mb female assembly and the 880.5 Mb male assembly is indicated by a contig length N50 of at least 58.6 Mb. BUSCO single-copy and duplicated completeness scores were 99.0% and 0.9% for the female assembly and 97.4% and 2.1% for the male assembly. Gene modeling identified at least 15,403 gene models in each genome with an average transcript length of 1.6 kb. Comparative analyses with other Onthophagini genomes indicated a dramatic expansion of repetitive sequences, which now comprise over 75% of this species’ genome and have driven the expansion of overall genome size to nearly twice that of close relatives. We combined the best-assembled chromosome-scale scaffolds from each sex to generate a hybrid reference assembly for this species. Comparative genomic analyses show that the nine autosomes and the X chromosome identified here in *O. binodis* are likely conserved throughout Onthophagini. Our sex-specific sequencing approach allowed us to identify putative Y chromosome sequences in the male assembly via coverage mapping and *k*-mer abundance comparisons. These genomes will be of great value to the scientific community as resources for studying insect genome evolution, development, and ecology.

Significance *Onthophagus binodis*, the humpbacked dung beetle, is a species of coprophagous beetle in the clade Onthophagini, which has worldwide agricultural importance and serves as an emerging model clade for research on the development and evolution of life history traits, evolutionary novelties, nutrition-sensitive growth, and sex differences. Genomic resources have been developed for other onthophagine species, but the full complement of sex chromosomes has not been identified to date for any species in this clade. Here, we report chromosome-level male and female genomes and putative sex chromosomes therein for *O. binodis*, which provide a new genomic resource for the study of sex differences in this hyperdiverse clade as well as studies of the evolution of coleopteran sex chromosomes more generally.

## Introduction

The humpbacked dung beetle *Onthophagus binodis* (Thunberg 1818) is a member of the geographically widespread and enormously speciose subfamily Scarabaeinae, also known as the true dung beetles. Like most members of the Onthophagini tribe (>2,500 species), *O. binodis* is obligately reliant on herbivore dung throughout its life cycle and thus is often found in close proximity to above-ground dung pads, where males compete for access to mating opportunities and females tunnel underground to lay eggs inside brood balls consisting of dung processed for larval food provisioning (Cook 1990). While *O. binodis* is native to sub-Saharan Africa, it is now also established in agricultural areas in Australia and Hawaii following a series of controlled introductions to mitigate fly pest populations due to the ability of beetle populations to efficiently process large quantities of manure, thereby cycling nutrients underground (Tyndale-Biscoe 1990, Markin and Yoshioka 1998). Accordingly, this species has served as an important model in agricultural research, management, and human–environment interactions.

In addition to their agricultural importance, *O. binodis* also emerged as an important study system in evolutionary biology, developmental biology, and behavioral ecology. Specifically, onthophagine beetles such as *O. binodis* are well recognized for the varied and often extreme exaggerated male weaponry used in competition over mates, diverse patterns of sexual dimorphism, and extraordinarily varied degree of nutritional plasticity (Moczek and Emlen 2000). As such, onthophagine species including *O. binodis* have emerged as powerful models for the study of diverse biological phenomena, including alternative reproductive tactics (Cook 1990), life history evolution (Simmons and Kotiaho 2007, Schwab et al. 2017), the origin of novel traits (Hu et al. 2019), and host–symbiont interactions (Rohner and Moczek 2021, Burdine et al. 2024).

Genomic resources have recently been developed for three closely related onthophagine species: *Onthophagus taurus*, *Onthophagus sagittarius*, and *Digitonthophagus gazella* (Davidson and Moczek 2024). These existing chromosome-level assemblies paved the way for comparative genomic and molecular analyses toward a better understanding of evolution and development in this clade but are agnostic to one important aspect of dung beetle genome evolution: the putative Y chromosome. Additionally, *O. binodis* itself exhibits a unique suite of traits of key interest in studies on the origin and diversification of novel complex traits: males possess a conspicuous thoracic horn—a trait that has diversified in intriguing ways within Onthophagini (Moczek et al 2006, Kijimoto et al. 2010)—but have lost head horns, which are ancestral to the clade (Emlen et al. 2005). Thus, establishing sex-specific genomic resources for this species will pave the way for future studies of convergent evolution and trait loss, as well as support future ecological and agricultural research involving this species. Here, we report independently assembled, chromosome-level annotated male and female genomes for *O. binodis*, which will serve as a powerful resource for dung beetle researchers and the broader insect research community.

## Results and Discussion

### Structure

The assembled female genome was 950.53 Mb, including a contig and scaffold N50 of 62.14 Mb, and 110.37 Mb, respectively. The male assembly was 880.47 Mb in length with a contig and scaffold N50 of 58.69 Mb and 102.98 Mb, respectively (see Table 1 for additional details on genome structure). However, chromosome-length scaffolds amount to 843.00 Mb in the female assembly and 814.44 Mb in the male and match the predicted number of autosomes and sex chromosomes for *Onthophagus* from karyotyping data (Wilson and Angus 2005). *K*-mer frequency analysis from the sequencing data predicted a genome size of 838 Mb and 831 Mb for the female and male samples (Fig. S5), suggesting the chromosomal scaffolds of the female comprise a highly complete representation of the genome, whereas the male assembly fell slightly short on this metric. Furthermore, telomere sequence analysis identified putative telomeric regions in seven chromosomal scaffolds of the female and in six scaffolds of the male assembly (Fig. S4). Lastly, BUSCO single-copy complete scores reached 98.1% and 95.3% in the female and male, respectively (see Table 1 complete BUSCO statistics). Taken together, these results indicate high contiguity and assembly completeness has been achieved for this species, particularly in the female assembly.

**Table 1:** *O. binodis* female (ObinF1.0) and male (ObinM1.0) genome assembly statistics compared to *O. taurus* (Otau3.0) assembly

	ObinF1.0	ObinM1.0	Otau3.0
Assembly			
Assembly size (Mb)	950.53	880.47	290.862
No. of scaffolds	132	97	34
N50 scaffold length (Mb)	110.37	102.98	33.174
Longest scaffold (Mb)	131.54	118.32	44.245
No. of scaffolds > 10 Mb	10	11	9
No. of contigs	159	132	45
N50 contig length	62.14	58.69	33.052
GC (%)	34.34	34.05	33.44
BUSCO (insecta_odb10, *n* = 1367)			
Complete	99.0 (99.0)	97.4 (97.4)	98.0 (98.0)
Single-copy complete (chromosomes only)	98.1 (98.1)	95.3 (95.3)	97.5 (97.5)
Duplicated	0.9 (0.9)	2.1 (2.1)	0.5 (0.5)
Fragmented	0.6 (0.6)	0.4 (0.4)	0.1 (0.1)
Missing	0.4 (0.4)	1.9 (1.9)	2.4 (2.4)
Annotations			
Number of genes	15,967	15,403	14,016
Number of transcripts	17,879	17,273	22,266
Transcripts per gene	1.12	1.12	1.59
BUSCO complete %	97.6	95.5	97.6
BUSCO single-copy complete %	94.1	92.8	96.6
BUSCO duplicated %	3.5	2.7	1.0
BUSCO fragmented %	0.4	0.4	0.1
BUSCO missing %	2.0	4.0	2.3
No. of introns	65,554	64,464	134,572
Introns per transcript	3.67	3.73	6.04
Average coding length (bp)	1,630	1,643	2,129
Median coding length (bp)	1155	1164	1410

Assembly statistics (top), assembly BUSCO analysis (middle), and annotation statistics (bottom) reported here cover all the scaffolds, and additional BUSCO analyses performed only on the assembled chromosomes are shown in parentheses. All BUSCO analyses were performed using the insecta_obd10 database (*n* = 1367).

While the male assembly is still largely represented by chromosome-length scaffolds, possible factors contributing to this assembly's less-complete result may be attributed to lower sequencing depth (∼17% less than the female) and slightly elevated heterozygosity, predicted from *k*-mer frequency data (1.26% vs. 0.97%). Although the female genome was better assembled overall, we detected assembly issues on Chr 10, as evidenced by unusual female:male read coverage patterns and a lack of identifiable telomeric sequence (Fig. S4). In order to generate an optimized reference assembly, we also present a “hybrid” genome composed the female X (chr1), female chr2-9, the male chr 10, and putative Y chromosomes (see below) for future analyses. The most surprising aspect of genome structure in this species was the overall size of the assemblies, nearly double the size of the next largest assembly of a related species (*O. sagittarius*: 553.3 Mb). This unusually large genome size can be explained almost entirely by the expansion of repetitive elements, which comprise 77.78% of the female assembly and 76.51%% of the male assembly (Fig. S2 and Data S1), proportions much higher than the 38.7% repetitive content in the *O. taurus* genome.

### Contamination

FCS-GX identified a total of 257 contigs containing significant amounts of non-Insecta DNA: 45 from the female assembly and 212 from the male. All identified contigs were removed, except for 11 from the male assembly that had less than 40% alignment coverage to contamination matches.

### Mitogenome

MitoHiFi identified three potential mitogenomes in the female assembly but only one in the male (unplaced_scaffold60). Of the three obtained from the female assembly, only one was circular and was the most similar to the reference mitogenomes of other *Onthophagus* species in both length and number of genes and was therefore selected (unplaced_scaffold41). Specifically, the circular female mitogenome sequence was 27,362 base pairs long and contained 37 genes, while the male mitogenome was not circular, 27,926 base pairs long, and also contained 37 genes. The female mitogenome was higher quality and was retained to represent the final mitogenome (Data S2). Finally, the mitogenome and associated gene predictions were rotated to begin at cytochrome c oxidase I (COX1).

### Gene Annotation

The program GALBA (Brůna et al. 2023) was used as the primary tool to predict gene models in the male and female genomes (Table 1). In the female genome, we identified 15,967 genes with an average coding length of 1.63 kb. On average, 17.6 genes per Mb were annotated across each chromosome. However, this metric varied from 22.2 (chromosomes 1(X) and 2) to 15.8 (chromosome 8) (Fig. S2 and Table S2), indicating a fairly even distribution of gene content throughout the genome. From these 15,967 gene models, 17,879 transcripts were predicted, averaging 1.12 transcripts per gene. Of these transcript models, 99.09% had significant hits to *O. taurus* models.

In the male genome, 15,403 genes were annotated, with an average coding length of 1.64 kb. On average, 16.6 genes per Mb were annotated across each chromosome. However, this metric varied from 22.9 (chromosome 2) to 16.1 (chromosome 8) (Fig. S2 and Table S2), again indicating a roughly equal distribution of genic content across the male genome. From the 15,403 gene models, 17,273 transcripts were predicted, again averaging 1.12 transcripts per gene. 99.02% of the transcripts had significant hits to *O. taurus* transcript models.

To compare outputs of two popular gene prediction tools, BRAKER was used to independently predict gene models in the two genomes (Gabriel et al. 2024). Those results are reported in the Supplementary material (Table S1). However, the best set of predictions (as evidenced by longer, more structurally complex gene models) resulted from the GALBA pipeline using gene models from the closely related species *O. taurus* as hints, in line with findings reported by Brůna et al. (2025), which found that GALBA outperforms BRAKER in the absence of RNA-sequencing data; thus, the GALBA annotations served as the final set used for all subsequent analyses.

### Synteny

**Fig. 1.**
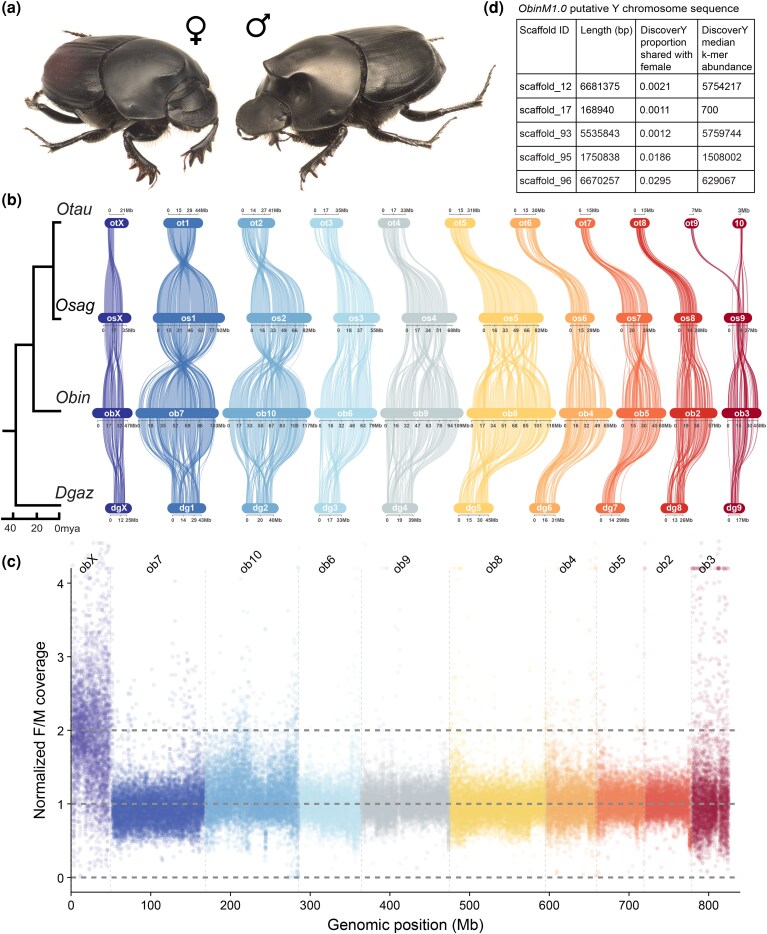
*Onthophagus binodis* genome assemblies offer insight into scarab sex chromosomes. a) *O. binodis* adult female (left) and adult male (right). b) Synteny analysis of the *O. binodis* hybrid genome with three other onthophagine genomes (data from Davidson and Moczek 2024) showing high degrees of chromosome-level synteny across the clade, with a conserved X chromosome and nine conserved autosomes. Note that the split of Ot chromosomes 9 and 10 (top row) is likely an artifact of the genome assembly process given past karyotype data indicating nine autosomes in this species (Wilson and Angus 2005). c) Read coverage plot showing the ratio of female/male sequencing reads mapped to the hybrid genome, establishing 2× coverage of the X chromosome in females. Each dot represents a 10 kb bin. d) Putative Y chromosome sequence within the male genome assembly identified by DiscoverY.

Analyses of conserved gene order were conducted to compare the genomes assembled here to recently published genomes for three closely related species, *O. taurus*, *O. sagittarius*, and *D. gazella* (Davidson and Moczek 2024). Previous karyotype analysis of many Onthophagini species, including *O. taurus* and *D. gazella*, established the presence of nine autosomal chromosomes, along with female homogametic and male heterogametic (XX/XY) sex chromosomes in these species (Wilson and Angus 2005). The genomes assembled here for *O. binodis* recapitulate the expected nine autosomes, which exhibit broad levels of synteny with their homologs in *O. taurus*, *O. sagittarius*, and *D. gazella* (Fig. 1a). There is no evidence of any fusion, fission, or translocation among the nine autosomal groups, although within each chromosome there appears to be a moderate number of inversions and translocations within the chromosome arms (Fig. 1a). The synteny of sex chromosomes is discussed below.

### Sex Chromosomes

Previous genomic analyses in *O. taurus*, *O. sagittarius*, and *D. gazella* mapped an intact X chromosome found to be largely syntenic with and thus homologous to the *Tribolium castaneum* X (Davidson and Moczek 2024). Synteny analyses performed on the genomes assembled here revealed conservation of this X chromosome in *O. binodis.* Mapping of the ratio of female/male coverage from PacBio long reads onto the hybrid assembly showed an expected 2× coverage of the X chromosome in females relative to males (Fig. 1c). To identify potential Y chromosome sequence within the ObinM1.0 assembly, we parsed the results of the coverage analysis and the DiscoverY analysis for small unplaced scaffolds with low read coverage ratio values and low proportion shared with the female genome, respectively (Table S3). We also filtered the list for scaffolds of total length below 10 Mb, as many published karyotypes from the genus *Onthophagus* denote a “punctiform” or extremely small Y chromosome relative to the size of the X (Blackmon and Desmuth 2015). This filtering approach resulted in a list of five small, unplaced scaffolds which we propose as putative Y chromosome sequences (Fig. 1d). Together, these results directly confirm female homogamety and suggest evidence for male heterogamety in this species. Y chromosomes can be particularly difficult to assemble, as they are predicted to degenerate rapidly through gene loss and repetitive element expansion due to the absence of recombination (Bachtrog 2013). We interpret these data to suggest that the sex chromosomes uncovered here are likely ancient within this clade of beetles, and thus, the *Onthophagus* Y has undergone extensive gene loss and degeneration, obscuring gene-level synteny across species.

## Materials and Methods

### Tissue Collection and DNA Sequencing

*Onthophagus binodis* adults were collected from cattle pastures in Waimea, Hawaii, in 2016. Colonies were continually maintained in sand-soil enclosures at Indiana University, Bloomington, United States, as described in Moczek and Nagy (2005). Inbreeding in this laboratory setting occurred for at least 20 generations before sample collection, theoretically reducing heterozygosity within the captive population. To collect tissue for high molecular weight DNA extraction and proximity ligation assays, whole late-stage male and female pupae were surface-sterilized with 70% ethanol, flash-frozen in liquid nitrogen, and stored at −80 °C. For PacBio HiFi sequencing, samples were shipped to the University of California-Davis DNA Technologies Core for long-read library preparation. Male and female libraries were sequenced on a single PacBio CCS Revio flow cell to generate approximately 52 Gb and 61 Gb of HiFi reads, respectively. For Hi-C proximity ligation, flash-frozen male and female pupae were (separately) ground in liquid nitrogen using a sterile mortar and pestle. Immediately following this step, ground tissue was treated with a formaldehyde solution to cross-link chromatin interactions, and the sample was further processed according to the Arima Genomics (Carlsbad, CA, United States) Hi-C kit protocol at the Indiana University Center for Computational Genomics and Bioinformatics sequencing core facility. For both the male and female samples, approximately 65 million 50 bp paired-end reads were sequenced on an Illumina NextSeq 2000 platform. Genome size and heterozygosity were estimated using GenomeScope v. 2.0 (Ranallo-Benavidez et al. 2020) using a *k*-mer length of 31. *K*-mer abundances were measured from the PacBio HiFi reads using Jellyfish v.2.3.1 (Marçais and Kingsford 2011).

### Assembly Strategy

HiFi PacBio sequencing data were used to generate independent male and female de novo contig-level genome assemblies. For each sex, HiFi reads were assembled into contigs using Hifiasm v0.19.9 (Cheng et al. 2021), and the completeness of each assembly was evaluated using BUSCO v5.7.1 (Simão et al. 2015 ) with the insecta_obd10 database. The female genome was assembled with default Hifiasm parameters, whereas the male genome was assembled with a modified -s parameter (*s* = 0.45), which controls the similarity threshold for purging duplicate haplotigs (Fig. S1). This parameter was modified due to a higher-than-expected proportion of duplicated single-copy orthologs (∼10%) under the default parameter setting (*s* = 0.55). To do so, we lowered the “s” parameter stepwise until an optimal “s” value was identified, as determined by BUSCO single-copy complete and duplication scores as well as overall genome size.

The contig assemblies were decontaminated using NCBI's Foreign Contamination Screen (FCS-GX) (Astashyn et al. 2024) run on Galaxy (The Galaxy Community 2024). Here, all contigs with significant alignment to taxonomic groups outside of Insecta not identified as common insertions in eukaryotic genomes were identified and removed. Lastly, Hi-C reads for both males and females were aligned to the contig assemblies using BWA (Li and Durbin, 2009). From these alignments, Hi-C contact maps and subsequent scaffolded assembly were generated with Juicer (Durand et al. 2016) and 3dDNA (Dudchenko et al. 2017), which was finalized using Juicebox (Robinson et al. 2018) (Fig. S6).

### Identification of Repetitive Elements and Coding Sequence

Repetitive elements in both genome assemblies were identified and quantified using the EarlGrey v.5.1.0 pipeline (Baril et al. 2024), which implements RepeatModeler (Flynn et al. 2022) and RepeatMasker alongside the Dfam Arthropoda library repetitive element library (Hubley et al. 2016: release 3.7). Repetitive regions throughout each genome assembly were subsequently soft-masked prior to the gene annotation process. We implemented *tidk* v0.2.65 (Brown et al. 2025) to identify putative telomeric sequence within chromosome-length scaffolds under default parameters (Fig. S4).

Gene models from the closely related species *O. taurus* were retrieved from Davidson and Moczek (2024) and incorporated into de novo gene prediction pipelines BRAKER v.3.0.8 (Gabriel et al. 2024) and GALBA v.1.0.11 (Brůna et al. 2023) for coding sequence predictions in the *O. binodis* male and female assemblies. The results of these two pipelines are presented in Table S1. The gene structures predicted by GALBA were selected as the final set of gene models for this species because this pipeline predicted longer transcript isoforms while maintaining overall proteome completeness as determined by BUSCO analysis (see below). Assembled protein models were functionally annotated using BlastP v2.6.0 (Camacho et al. 2009) against the *O. taurus* protein models from Davidson and Moczek (2024) and annotated by NCBI Eukaryotic Genome Annotation Pipeline. Finally, BUSCO v3 (Seppey et al. 2019) was used to analyze the completeness of an evolutionarily-informed gene set of 1,367 putatively ancestral genes for the class Insecta within the genome assemblies (parameters: –db insecta_odb10).

### Mitogenome

Mitochondrial genomes were identified from both female and male *O. binodis* assemblies using MitoHiFi v2 (Uliano-Silva et al. 2023) on Galaxy (The Galaxy Community 2024). MitoHiFi was run on pre-assembled contigs with the *Onthophagus fodiens* mitochondrion as a reference (PQ067330.1). Final gene annotations were conducted with MITOS2 v2.1.9 (Al Arab et al. 2017, Donath et al. 2019).

### Genomic Synteny Analysis

To compare the male and female assemblies generated here and to assess genome evolution across the Onthophagini, MCScanX was used to detect collinear blocks of genes (Wang et al. 2024) in the male and female assemblies (Fig. S3), as well as between the *O. binodis* male genome and the genomes of *O. taurus*, *O. sagittarius*, and *D. gazella* (Fig. 1b). The MCScanX results were imported into SynVisio to visualize chromosomal reorganizations across these genomes, including duplication, inversion, and translocation of collinear gene blocks (Bandi and Gutwin 2020).

### Sex Chromosome Identification

To identify putative sex-linked chromosomes, we compared the positional coverage of female and male reads when aligned to the final male assembly. Raw HiFi reads from both sexes were mapped to the male assembly using minimap2 v2.28 (Li 2018) and filtered for a mapping quality score of 30. Coverage was calculated with bamCoverage in deepTools v3.5.6 (Ramírez et al. 2016) using a bin size of 10 kb and normalized to counts per million. Coverage in each bin was further normalized by the median coverage of all bins in each sex-specific alignment, and the female-to-male coverage ratio was calculated for each chromosome to identify nine autosomes, as well as putative X and Y chromosomes (Table S3). As an additional measure to attempt to identify male-specific contigs, we ran DiscoverY using the options “–female_kmers_set –kmer_size 31 –female_bloom_capacity 24000000 –mode female + male” (Rangavittal et al. 2019). The female *k*-mer set required for DiscoverY was generated with DSK (Rizk et al. 2013) using the assembled female genome FASTA file.

## Supplementary Material

evag023_Supplementary_Data

## Data Availability

Data associated with these projects have been deposited on NCBI: Genome assembly and related sequencing files for the female and male individuals are available under the BioProject PRJNA1308620 and PRJNA1308621, respectively. Repetitive element annotation results are available in Data S1. Mitochondrial genome annotation results are available in Data S2. Genome annotation files including gene and protein models and their genomic loci for the female and male assemblies are available on Figshare (10.6084/m9.figshare.30815540). Genome assemblies and sequencing data are available on NCBI under BioProject accessions PRJNA1308620 (female) and PRJNA1308621 (male).
