# Supplementary material for: Chromosome-Level Sex-Specific Genome Assemblies of Onthophagus binodis Provide Insight into Scarab Sex Chromosomes
Source: Genome Biol Evol. 2026 Jan 29;18(2):evag023. doi: 10.1093/gbe/evag023 (PMC12902608; doi:10.1093/gbe/evag023)
Supplement: evag023_Supplementary_Data [file evag023_supplementary_data.zip › Revised-supplement.docx]

**Supplementary Table 1:** Comparison of GALBA and BRAKER gene model prediction. BUSCO analysis was completed using the insecta_odb10 database (n=1367). Only the longest isoform for each gene model is considered for the BUSCO analyses.

| **Assembly** | **ObinF1.0** | | **ObinM.10** | | ***O. taurus*** |
| --- | --- | --- | --- | --- | --- |
| **Tool** | **BRAKER** | **GALBA** | **BRAKER** | **GALBA** | **NCBI** |
| **# Genes** | 20273 | 15967 | 19727 | 15403 | 14016 |
| **# Transcripts** | 24,138 | 17879 | 23604 | 17273 | 22266 |
| **Transcript:Gene** | 1.19 | 1.12 | 1.20 | 1.12 | 1.59 |
| ***BUSCO complete %*** | 97.0 | 97.6 | 96.0 | 95.5 | 97.6 |
| ***BUSCO single copy complete %*** | 93.6 | 94.1 | 93.2 | 92.8 | 96.6 |
| ***BUSCO duplicated %*** | 3.4 | 3.5 | 2.8 | 2.7 | 1 |
| ***BUSCO fragmented %*** | 1.5 | 0.4 | 1.0 | 0.4 | 0.1 |
| ***BUSCO missing %*** | 1.5 | 2.0 | 3.0 | 4.0 | 2.3 |
| **# Introns** | 64088 | 65554 | 64107 | 64464 | 134572 |
| **Introns:Transcript** | 2.66 | 3.67 | 2.72 | 3.73 | 6.04 |
| **Avg. coding length (bp)** | 1340 | 1630 | 1383 | 1643 | 2129 |
| **Median coding length (bp)** | 903 | 1155 | 933 | 1164 | 1410 |
| **% hits to *O. taurus* models** | 86.15 | 99.09 | 87.21 | 99.02 | NA |

**Supplementary Table 2:** Summary of chromosome lengths, gene count, repetitive sequence and gap (N) sequence for the *ObinF1.0 and ObinM1.0* assemblies. Data presented for contigs greater than N90 length.

| **Chr** | **Chromosome Length (Mb)** | **Gene Count** | **Repeat Length (Mb)** | **Repeat %** |
| --- | --- | --- | --- | --- |
| **ObinF1.0** | | | | |
| 1(X) | 48.94 | 1088 | 38.01 | 77.66 |
| 2 | 59.93 | 1333 | 48.47 | 80.87 |
| 3 | 47.02 | 932 | 38.75 | 82.40 |
| 4 | 63.86 | 1299 | 50.33 | 78.81 |
| 5 | 59.86 | 1060 | 46.78 | 78.15 |
| 6 | 79.02 | 1525 | 57.81 | 73.16 |
| 7 | 119.78 | 2075 | 86.69 | 72.38 |
| 8 | 122.68 | 1933 | 89.21 | 72.72 |
| 9 | 110.37 | 1966 | 81.81 | 74.13 |
| 10 | 131.54 | 2398 | 96.60 | 73.44 |
| rest | 107.53 | 358 | 104.58 | 97.51 |
| Total | 950.53 | 15967 | 739.33 | 77.78 |
| **ObinM1.0** | | | | |
| 1(X) | 46.87 | 1037 | 36.37 | 77.61 |
| 2 | 56.63 | 1294 | 45.43 | 80.22 |
| 3 | 44.51 | 877 | 36.52 | 82.04 |
| 4 | 64.98 | 1330 | 51.50 | 79.25 |
| 5 | 59.84 | 1051 | 47.01 | 78.57 |
| 6 | 79.19 | 1511 | 58.31 | 73.63 |
| 7 | 102.98 | 1819 | 72.41 | 70.32 |
| 8 | 118.32 | 1907 | 85.27 | 72.06 |
| 9 | 109.70 | 2003 | 81.50 | 74.29 |
| 10 | 116.96 | 2140 | 84.90 | 72.59 |
| rest | 80.94 | 434 | 63.40 | 96.03 |
| Total | 880.47 | 15403 | 662.63 | 75.26 |

**Supplementary Table 3:** Identification of putative Y chromosomes within ObinM1.0 via DiscoverY and read coverage ratio analysis.

| **Scaffold** | **Length (bp)** | **DiscoverY proportion shared with female** | **DiscoverY median k-mer abundance** | **median scaffold read coverage - male** | **median scaffold read coverage - female** | **Read coverage ratio female/**  **male** |
| --- | --- | --- | --- | --- | --- | --- |
| chr1 | 46868762 | 0.4214 | 86 | 0.4743 | 0.9827 | 2.0717 |
| chr2 | 56628615 | 0.5377 | 67 | 1.0003 | 0.9740 | 0.9738 |
| chr3 | 44513771 | 0.4791 | 109 | 0.9281 | 1.0088 | 1.0870 |
| chr4 | 64983765 | 0.5960 | 60 | 1.0518 | 1.0523 | 1.0005 |
| chr5 | 59838209 | 0.5877 | 60 | 1.0415 | 1.0262 | 0.9853 |
| chr6 | 79191960 | 0.6257 | 61 | 1.0518 | 0.9827 | 0.9343 |
| chr7 | 102981365 | 0.6738 | 47 | 1.0621 | 1.0088 | 0.9498 |
| chr8 | 118318894 | 0.6492 | 60 | 1.0518 | 1.0001 | 0.9509 |
| chr9 | 109704904 | 0.6140 | 66 | 1.0106 | 0.9827 | 0.9724 |
| chr10 | 116960297 | 0.6369 | 65 | 1.0106 | 1.0697 | 1.0585 |
| scaffold_12 | 6681375 | 0.0021 | 5754217 | 0.0825 | 0.0391 | 0.4744 |
| scaffold_13 | 36536 | 0.9114 | 2 | 0.1598 | 0.0696 | 0.4353 |
| scaffold_14 | 78465 | 0.0027 | 5759744 | 0.0000 | 0.0000 | NA |
| scaffold_15 | 50642 | 0.0969 | 9555 | 0.0000 | 0.0000 | NA |
| scaffold_16 | 77379 | 0.0925 | 9551 | 0.0000 | 0.0000 | NA |
| scaffold_17 | 168940 | 0.0011 | 700 | 0.0309 | 0.0174 | 0.5622 |
| scaffold_18 | 816365 | 0.0031 | 5763286 | 0.0000 | 0.0000 | NA |
| scaffold_19 | 501268 | 0.6543 | 511 | 0.1134 | 0.0000 | 0.0000 |
| scaffold_20 | 441205 | 0.0010 | 5758347 | 0.0361 | 0.0783 | 2.1686 |
| scaffold_21 | 780157 | 0.0028 | 5763286 | 0.0000 | 0.0000 | NA |
| scaffold_22 | 219232 | 0.0022 | 5763286 | 0.0000 | 0.0000 | NA |
| scaffold_23 | 73205 | 0.0937 | 9562 | 0.0000 | 0.0000 | NA |
| scaffold_24 | 70157 | 0.0962 | 9541 | 0.0000 | 0.0000 | NA |
| scaffold_25 | 65839 | 0.0123 | 9545 | 0.0000 | 0.0000 | NA |
| scaffold_26 | 65804 | 0.0910 | 9555 | 0.0000 | 0.0000 | NA |
| scaffold_27 | 65564 | 0.9117 | 3 | 0.0722 | 0.0000 | 0.0000 |
| scaffold_28 | 63817 | 0.0037 | 5764554 | 0.0000 | 0.0000 | NA |
| scaffold_29 | 62193 | 0.0000 | 9562 | 0.0000 | 0.0087 | Inf |
| scaffold_30 | 61941 | 0.1010 | 9544 | 0.0000 | 0.0043 | Inf |
| scaffold_31 | 61479 | 0.0146 | 9538 | 0.0000 | 0.0043 | Inf |
| scaffold_32 | 60731 | 0.0878 | 9578 | 0.0000 | 0.0000 | NA |
| scaffold_33 | 60383 | 0.1234 | 9538 | 0.0000 | 0.0000 | NA |
| scaffold_34 | 58210 | 0.1040 | 9536 | 0.0000 | 0.0043 | Inf |
| scaffold_35 | 57026 | 0.1028 | 9541 | 0.0000 | 0.0000 | NA |
| scaffold_36 | 53667 | 0.0000 | 5773279 | 0.0000 | 0.0000 | NA |
| scaffold_37 | 53597 | 0.0059 | 9537 | 0.0000 | 0.0000 | NA |
| scaffold_38 | 53171 | 0.0023 | 9549 | 0.0052 | 0.0043 | 0.8434 |
| scaffold_39 | 52592 | 0.0142 | 9567 | 0.0000 | 0.0087 | Inf |
| scaffold_40 | 52386 | 0.0947 | 9555 | 0.0000 | 0.0000 | NA |
| scaffold_41 | 47936 | 0.0668 | 9560 | 0.0000 | 0.0000 | NA |
| scaffold_42 | 47710 | 0.1049 | 9551 | 0.0000 | 0.0000 | NA |
| scaffold_43 | 47615 | 0.0125 | 9559 | 0.0103 | 0.0000 | 0.0000 |
| scaffold_44 | 47605 | 0.0721 | 9555 | 0.0000 | 0.0000 | NA |
| scaffold_45 | 46479 | 0.0024 | 5763907 | 0.0000 | 0.0000 | NA |
| scaffold_46 | 46244 | 0.9104 | 4 | 0.0722 | 0.0000 | 0.0000 |
| scaffold_47 | 45679 | 0.9115 | 4 | 0.2836 | 0.0783 | 0.2760 |
| scaffold_48 | 44632 | 0.1039 | 9549 | 0.0052 | 0.0000 | 0.0000 |
| scaffold_49 | 44164 | 0.9096 | 3 | 0.0464 | 0.0174 | 0.3748 |
| scaffold_50 | 43480 | 0.9090 | 2 | 0.0516 | 0.0348 | 0.6747 |
| scaffold_51 | 43054 | 0.0024 | 40911 | 0.4022 | 0.2783 | 0.6920 |
| scaffold_52 | 41558 | 0.0244 | 1862.5 | 0.0155 | 0.0043 | 0.2811 |
| scaffold_53 | 41161 | 0.0066 | 9530 | 0.0000 | 0.0000 | NA |
| scaffold_54 | 39161 | 0.0946 | 9556 | 0.0000 | 0.0043 | Inf |
| scaffold_55 | 38190 | 0.9103 | 4 | 0.0619 | 0.0000 | 0.0000 |
| scaffold_56 | 37629 | 0.9115 | 3 | 0.0619 | 0.0000 | 0.0000 |
| scaffold_57 | 31985 | 0.9095 | 3 | 0.0516 | 0.0000 | 0.0000 |
| scaffold_58 | 29624 | 0.9086 | 2 | 0.0309 | 0.0043 | 0.1406 |
| scaffold_59 | 29143 | 0.0674 | 13421 | 0.1031 | 0.0522 | 0.5060 |
| scaffold_60 | 27926 | 0.0000 | 691.5 | 0.2784 | 0.9566 | 3.4359 |
| scaffold_61 | 26205 | 0.0936 | 9556 | 0.0000 | 0.0000 | NA |
| scaffold_62 | 24844 | 0.8781 | 89 | 0.5156 | 0.0609 | 0.1181 |
| scaffold_63 | 23348 | 0.9096 | 4 | 0.0825 | 0.0000 | 0.0000 |
| scaffold_64 | 20027 | 0.9123 | 3 | 0.0412 | 0.0000 | 0.0000 |
| scaffold_65 | 365558 | 0.0009 | 5771391 | 0.0000 | 0.0000 | NA |
| scaffold_66 | 1781020 | 0.0007 | 5759744 | 0.0103 | 0.0087 | 0.8434 |
| scaffold_67 | 279345 | 0.0002 | 5764554 | 0.0000 | 0.0000 | NA |
| scaffold_68 | 1442371 | 0.0028 | 5763286 | 0.0103 | 0.0478 | 4.6385 |
| scaffold_69 | 192308 | 0.0001 | 5754217 | 0.0000 | 0.0000 | NA |
| scaffold_70 | 50864 | 0.0038 | 9553 | 0.0000 | 0.0000 | NA |
| scaffold_71 | 39137 | 0.9083 | 4 | 0.1341 | 0.0565 | 0.4217 |
| scaffold_72 | 164987 | 0.0010 | 9539 | 0.3558 | 0.5740 | 1.6134 |
| scaffold_73 | 1221296 | 0.0184 | 1579515 | 0.2784 | 0.4609 | 1.6555 |
| scaffold_74 | 500000 | 0.0744 | 12703 | 0.5826 | 0.4740 | 0.8135 |
| scaffold_75 | 250000 | 0.0010 | 5759350 | 0.0000 | 0.0000 | NA |
| scaffold_76 | 125000 | 0.5277 | 68 | 0.9074 | 0.8697 | 0.9584 |
| scaffold_77 | 250000 | 0.0305 | 7345 | 0.5465 | 0.5218 | 0.9547 |
| scaffold_78 | 2875000 | 0.0020 | 5754217 | 0.1237 | 0.1305 | 1.0542 |
| scaffold_79 | 529953 | 0.0003 | 5763917 | 0.0000 | 0.0000 | NA |
| scaffold_80 | 125000 | 0.1964 | 2272 | 0.4847 | 0.2957 | 0.6101 |
| scaffold_81 | 125000 | 0.0029 | 4037419 | 0.0309 | 0.0087 | 0.2811 |
| scaffold_82 | 2225000 | 0.0008 | 5757103 | 0.0103 | 0.0087 | 0.8434 |
| scaffold_83 | 50000 | 0.0346 | 100104 | 0.5465 | 0.3174 | 0.5808 |
| scaffold_84 | 3353889 | 0.0018 | 5763286 | 0.0155 | 0.0130 | 0.8434 |
| scaffold_85 | 500000 | 0.7364 | 39 | 0.5672 | 0.0348 | 0.0613 |
| scaffold_86 | 531576 | 0.2108 | 801 | 0.4847 | 0.4783 | 0.9869 |
| scaffold_87 | 250000 | 0.1214 | 6773.5 | 0.4125 | 0.0391 | 0.0949 |
| scaffold_88 | 4334296 | 0.0152 | 1496131 | 0.2990 | 0.2783 | 0.9306 |
| scaffold_89 | 125000 | 0.0129 | 1600584 | 0.4331 | 0.0174 | 0.0402 |
| scaffold_90 | 125000 | 0.1537 | 16119 | 0.6393 | 0.5566 | 0.8706 |
| scaffold_91 | 9200000 | 0.0028 | 5759744 | 0.0103 | 0.0087 | 0.8434 |
| scaffold_92 | 125000 | 0.0212 | 662 | 0.5981 | 0.4957 | 0.8288 |
| scaffold_93 | 5535843 | 0.0012 | 5759744 | 0.0206 | 0.0087 | 0.4217 |
| scaffold_94 | 500000 | 0.0034 | 5727756 | 0.1444 | 0.1044 | 0.7229 |
| scaffold_95 | 1750838 | 0.0186 | 1508002 | 0.2784 | 0.1565 | 0.5622 |
| scaffold_96 | 6670257 | 0.0295 | 629067 | 0.4125 | 0.2870 | 0.6958 |
| scaffold_97 | 14456206 | 0.7043 | 39 | 0.5259 | 0.0087 | 0.0165 |
| scaffold_98 | 46030571 | 0.1615 | 1485602 | 0.3197 | 0.0174 | 0.0544 |
| scaffold_99 | 8573229 | 0.0014 | 5754217 | 0.0928 | 0.0696 | 0.7497 |


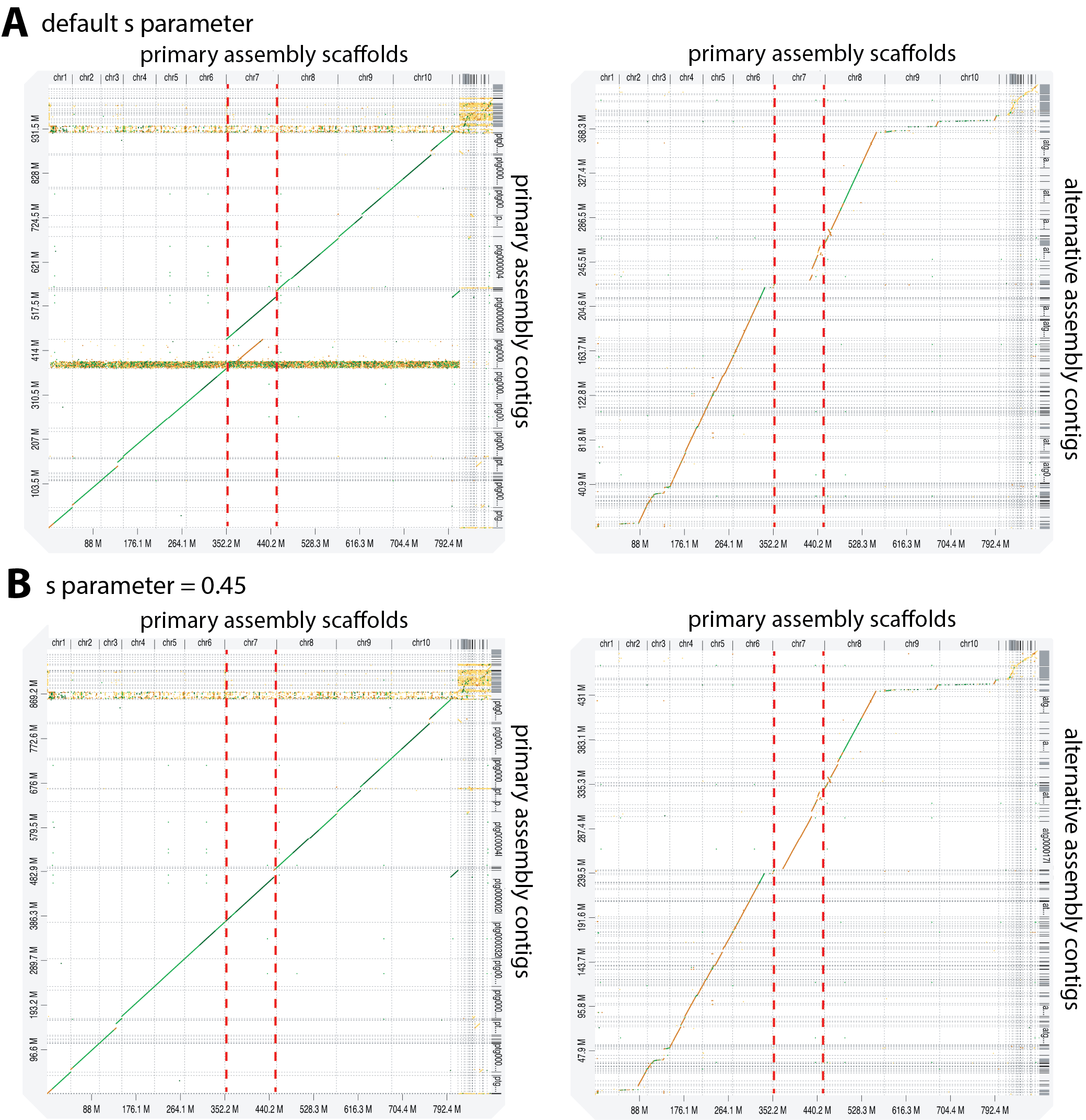


**Supplementary Figure 1:** Modification of *hifiasm* s parameter when assembling the ObinM1.0 genome

(A) D-GENIES genomic alignment dot plot (Cabanettes and Klopp 2018) mapping contigs from the hifiasm primary assembly (left) and the hifiasm alternative assembly (right) onto the fully scaffolded ObinM1.0 assembly under the default parameter s=0.55. Dotted red lines highlight a region of chromosome 7 with fairly diverged haplotypes such that both are included in the primary assembly. (B) After revising the hifiasm parameters to s=0.45, dot plots mapping contigs from the primary assembly (left) and from the alternative assembly (right) onto the fully scaffolded ObinM1.0 assembly show that only a single haplotype of chromosome 7 is included in the primary assembly (dotted red lines); this version of the assembly was used for downstream assembly and analysis.


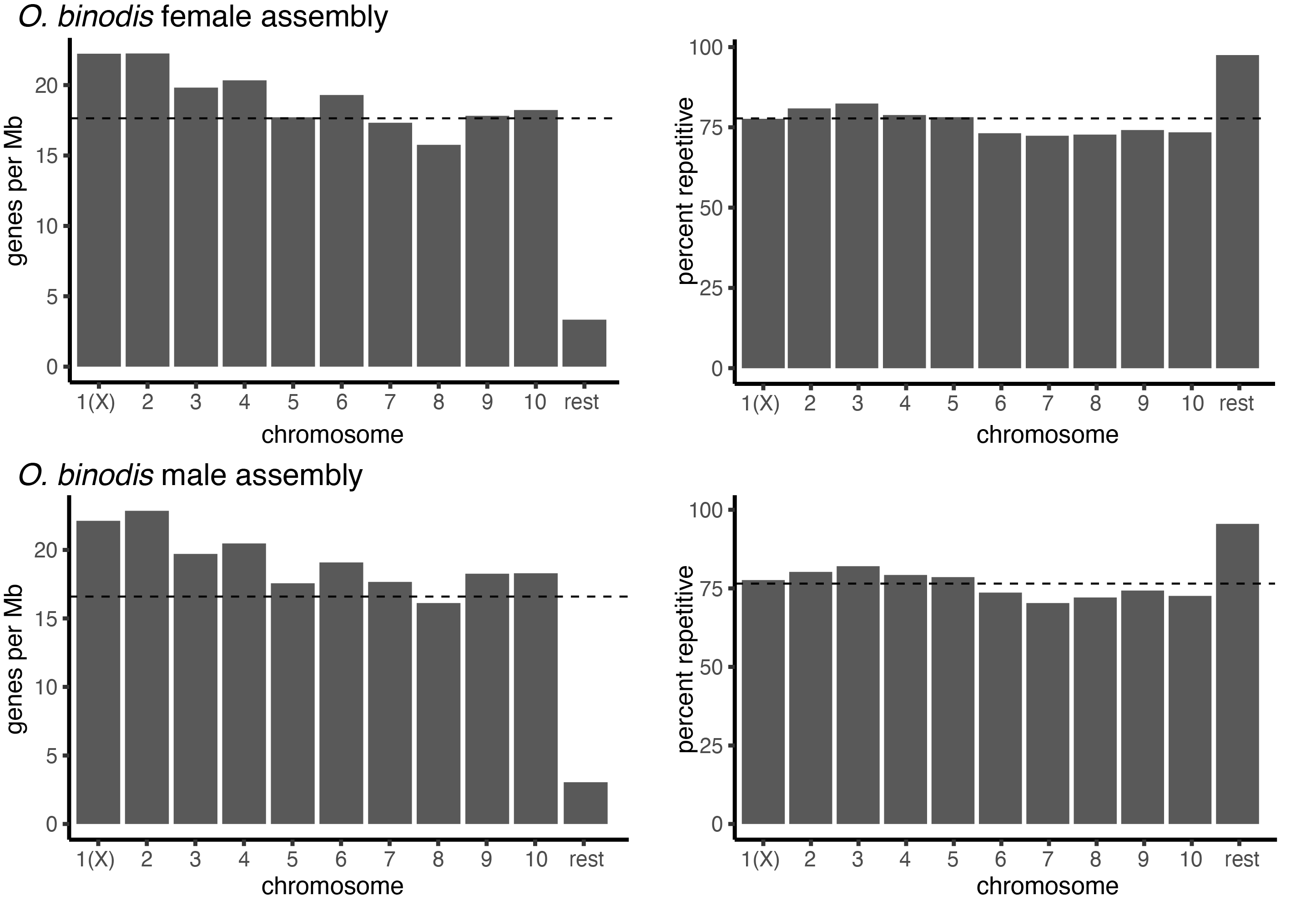


**Supplementary Figure 2:** Frequency of coding regions and repetitive regions across the genome assemblies.

(Left) Number of annotated genes per megabase (Mb) for each chromosome in the ObinF1.0 assembly (top) and ObinM1.0 assembly (bottom). Dotted lines mark the average number of genes per Mb across the entire genome (17.6 for the female assembly, 16.6 for the male assembly). (Right) Percentage of repetitive sequence comprising each chromosome in each assembly. Dotted lines mark the total percentage repetitive sequence the entire genome (77.78% for the female assembly, 76.51% for the male assembly).


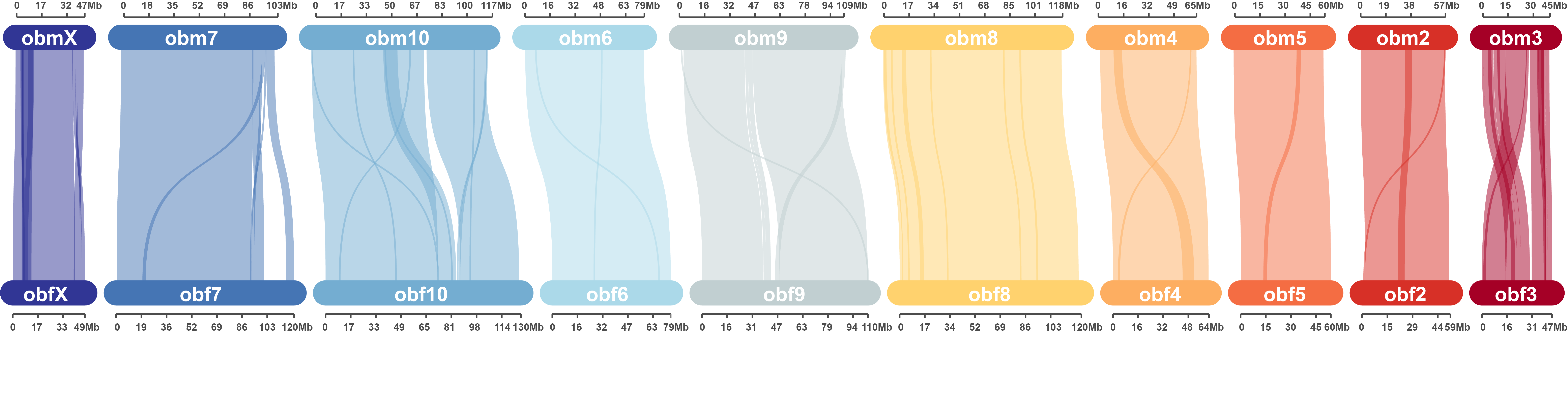
**Supplementary Figure 3:** Synteny between chromosomes in the ObinM1.0 and ObinF1.0 genome assemblies. The X chromosome and all autosomes show broad synteny between the male (top) and female (bottom) genome assembly.


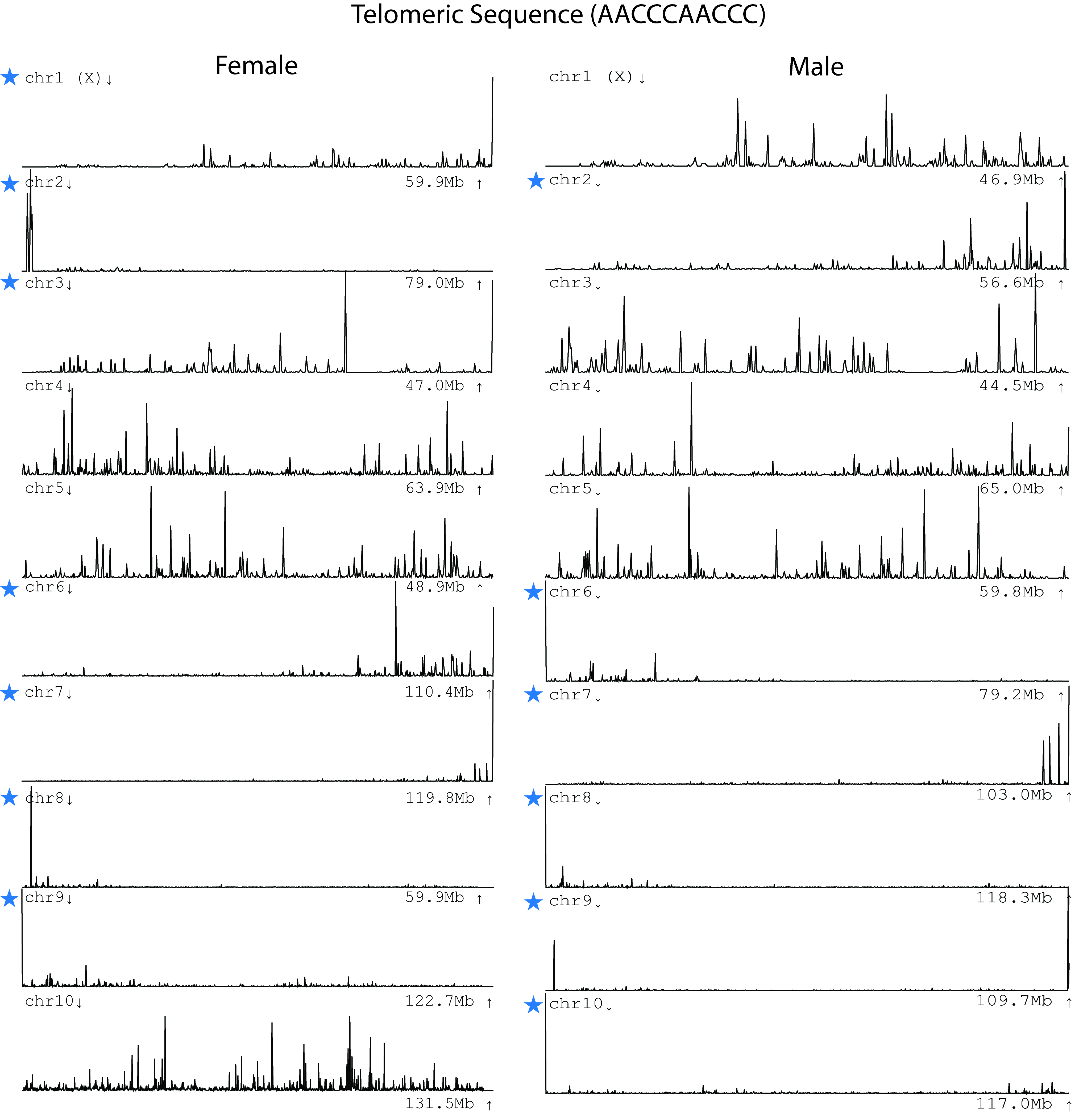


**Supplementary Figure 4:** Identification of putative telomeric sequence (AACCC) in female (left) and male (right) genome assemblies was performed using tidk v0.2.65 (Brown et al. 2025). When scanning assemblies for telomeres, a doublet of the telomeric sequence (AACCCAACCC) was used to improve signal-to-noise ratio across each scaffold. Telomere abundance (y-axis) is scaled to the maximum abundance for each scaffold, resulting in a noisy signal throughout scaffolds lacking identifiable telomeres (e.g., chromosomes 4 and 5). Blue stars denote scaffolds where telomeric sequence was identified at least on one terminal end, suggesting a largely complete representation of the chromosome was captured in the assembly process.


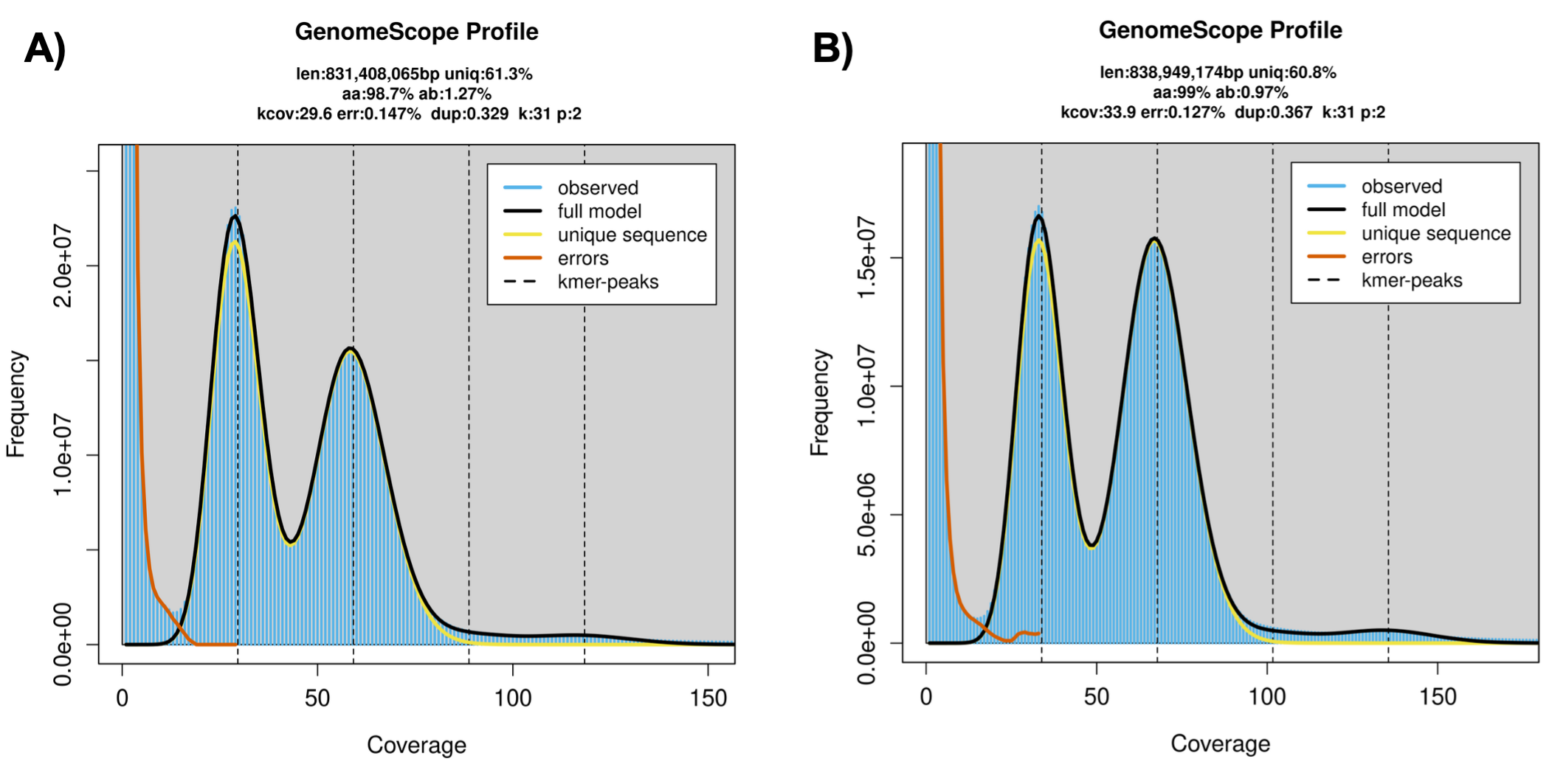


**Supplementary Figure 5:** GenomeScope v2.0 (Ranallo-Benavidez et al., 2020) profiles for *Onthophagus binodis* **A)** male and **B)** female individuals using k-mer (length = 31) abundances calculated by Jellyfish v2.3.1 from PacBio HiFi reads (Marçais and Kingsford, 2011). GenomeScope estimated assembly sizes of approximately 831 Mb for the male and 838 Mb for the female, with genome-wide heterozygosity rates of about 1.26% and 0.97%, respectively.





**Supplementary Figure 6:** HiC contact map of scaffolds from the female and male *Onthophagus binodis* genome assemblies before (left) and after (right) curation. The original boundaries in the “before” panels are recolored to green in the “after” panels.

**References**

Brown, Max R, Pablo Manuel Gonzalez de La Rosa, Mark Blaxter, tidk: a toolkit to rapidly identify telomeric repeats from genomic datasets, *Bioinformatics*, Volume 41, Issue 2, February 2025, btaf049

Cabanettes F, Klopp C. (2018) D-GENIES: dot plot large genomes in an interactive, efficient and simple way. PeerJ 6:e4958 <https://doi.org/10.7717/peerj.4958>

Marçais, Guillaume, Carl Kingsford. A fast, lock-free approach for efficient parallel counting of occurrences of *k*-mers, *Bioinformatics*, Volume 27, Issue 6, March 2011, Pages 764–770

Ranallo-Benavidez, T.R., Jaron, K.S. & Schatz, M.C. GenomeScope 2.0 and Smudgeplot for reference-free profiling of polyploid genomes. *Nat Commun* **11**, 1432 (2020).
